# Effects of the ventilatory stimulant, doxapram on human TASK‐3 (KCNK9, K2P9.1) channels and TASK‐1 (KCNK3, K2P3.1) channels

**DOI:** 10.1111/apha.13361

**Published:** 2019-09-18

**Authors:** Kevin P. Cunningham, D. Euan MacIntyre, Alistair Mathie, Emma L. Veale

**Affiliations:** ^1^ Medway School of Pharmacy University of Greenwich and University of Kent Chatham Maritime UK; ^2^ Department of Drug Discovery Galleon Pharmaceuticals, Inc Horsham Pennsylvania

**Keywords:** doxapram, enantiomers, heterodimers, K2P channels, respiratory stimulant, TASK‐1 channels, TASK‐3 channels

## Abstract

**Aims:**

The mode of action by which doxapram acts as a respiratory stimulant in humans is controversial. Studies in rodent models, have shown that doxapram is a more potent and selective inhibitor of TASK‐1 and TASK‐1/TASK‐3 heterodimer channels, than TASK‐3. Here we investigate the direct effect of doxapram and chirally separated, individual positive and negative enantiomers of the compound, on both human and mouse, homodimeric and heterodimeric variants of TASK‐1 and TASK‐3.

**Methods:**

Whole‐cell patch clamp electrophysiology on tsA201 cells was used to assess the potency of doxapram on cloned human or mouse TASK‐1, TASK‐3 and TASK‐2 channels. Mutations of amino acids in the pore‐lining region of TASK‐3 channels were introduced using site‐directed mutagenesis.

**Results:**

Doxapram was an equipotent inhibitor of human TASK‐1 and TASK‐3 channels, compared with mouse channel variants, where it was more selective for TASK‐1 and heterodimers of TASK‐1 and TASK‐3. The effect of doxapram could be attenuated by either the removal of the C‐terminus of human TASK‐3 channels or mutations of particular hydrophobic residues in the pore‐lining region. These mutations, however, did not alter the effect of a known extracellular inhibitor of TASK‐3, zinc. The positive enantiomer of doxapram, GAL‐054, was a more potent antagonist of TASK channels, than doxapram, whereas the negative enantiomer, GAL‐053, had little inhibitory effect.

**Conclusion:**

These data show that in contrast to rodent channels, doxapram is a potent inhibitor of both TASK‐1 and TASK‐3 human channels, providing further understanding of the pharmacological profile of doxapram in humans and informing the development of new therapeutic agents.

## 1 INTRODUCTION

Doxapram (1‐ethyl‐4‐(2‐morpholinoethyl)‐3,3‐diphenyl‐2‐pyrrolidinone) is a central respiratory stimulant used clinically in the treatment of post‐operative respiratory depression, acute respiratory failure, chronic obstructive pulmonary disorder and apnoea in premature infants.^1^, ^2^ Doxapram's analeptic respiratory action is characterized by an increase in tidal volume and a slight increase in respiratory rate, when administered intravenously.^3^ The compound's mode of action has long been debated, with conflicting data from animal and human models.^2^ Recent studies propose a mode of action that occurs via the direct stimulation of peripheral chemoreceptors of type 1 cells within the carotid bodies and a subsequent release of catecholamines and other neurotransmitters.^4^, ^5^ This results in the prevention or reversal of central nervous system depressant or narcotic–induced respiratory failure. Neurotransmitter release by type 1 cells occurs in response to an increase in cytosolic calcium (Ca^2+^) levels, mediated by voltage‐gated calcium channels in response to an electrical signal causing the membrane to depolarize.

A number of potassium (K) channels have been identified in type 1 cells, including delayed rectifier K^+^‐channels, calcium‐activated K‐channels, HERG channels and TWIK‐related acid‐sensitive K^+^‐channels (TASK).^6^, ^7^, ^8^, ^9^, ^10^ The resting membrane K conductance of carotid bodies, has been shown to be predominantly mediated by a TASK‐like current, which when inhibited, results in an influx of Ca^2+^ ions and subsequent membrane depolarization.^11^ Both TASK‐1 (KCNK3) and TASK‐3 (KCNK9) are expressed in the carotid body,^12^, ^13^ present as a mixture of homodimeric and heterodimeric TASK‐1 and TASK‐3 channels, with heterodimeric channels, the predominant form.^13^, ^14^ Stimulation of chemoreceptors by doxapram is thought to occur via the direct inhibition of a TASK channel.^15^, ^16^ Indeed, mice lacking either TASK‐1 or both TASK‐1 and TASK‐3 have impaired carotid body function.^17^, ^18^

Previous work,^15^ on cloned rat TASK channels, showed that doxapram's selectivity favoured TASK‐1 homodimeric channels, followed by TASK‐1/TASK‐3 heterodimeric channels and to a lesser extent TASK‐3 homodimeric channels, with EC_50_’s of 410 nM, 9 µM and 37 µM respectively. With a therapeutic range of 4‐5 µM for doxapram in the blood plasma,^19^ respiratory stimulation in the rat would appear to occur predominantly through homodimeric rat TASK‐1 channels. The differential selectivity between the channels was thought to reside at the carboxy intracellular domains of the channels, where homology between TASK‐1 and TASK‐3 sequence is the least.^15^

Later studies from a number of groups identified, using molecular modelling, a common intracellular binding site at the pore region of rat TASK channels that was thought to transduce the inhibitory effects of a number of related compounds, A1899, PKTHPP and doxapram.^20^, ^21^, ^22^ Four amino acids in the pore region of rat TASK‐3, Leucine (L) 122, Glycine (G) 236, L 239 and valine (V) 242, were shown to effect the efficacy of compounds such as doxapram, when mutated to an aspartate (D), highlighting the importance of this region in TASK channels for the action of these compound types.^22^ Interestingly, one of these highlighted amino acids, G 236 when mutated to an arginine (R) is responsible for the condition, Birk Barel mental retardation syndrome.^23^

Doxapram is a racemic compound which is comprised of positive (+) and negative (−) enantiomers. Galleon Pharmaceuticals showed that by chirally separating doxapram into its positive (+) (GAL‐054) and negative (−) (GAL‐053) enantiomers, that ventilatory stimulation was conferred by GAL‐054 and not GAL‐053. Moreover, the adverse events such as dysrhythmias, agitation and seizures, observed with doxapram, were only observed with GAL‐053.^24^, ^25^, ^26^, ^27^ Phase 1 trials with GAL‐054 in healthy volunteers, however, found that GAL‐054 caused hypertension, as previously seen in rat models.^27^

In this study, using electrophysiological techniques and heterologous cell expression systems we investigated the direct effect of doxapram on human and mouse cloned homodimeric and heterodimeric TASK channels and a structurally related channel, from the TALK subfamily, TASK‐2.

Also, we further investigated the mode of action of doxapram on human TASK‐3 channels, using pore specific mutations and C‐terminally truncated channels.

Finally, we studied the effect of positive and negative enantiomers of doxapram, GAL‐054 and GAL‐053, in isolation on human homodimeric TASK‐1 and TASK‐3 channels. A preliminary account of some of these data has been reported previously.^28^

## 2 RESULTS

### 2.1 Doxapram is a potent inhibitor of both TASK‐1 and TASK‐3 human cloned channels

**Figure 1:**
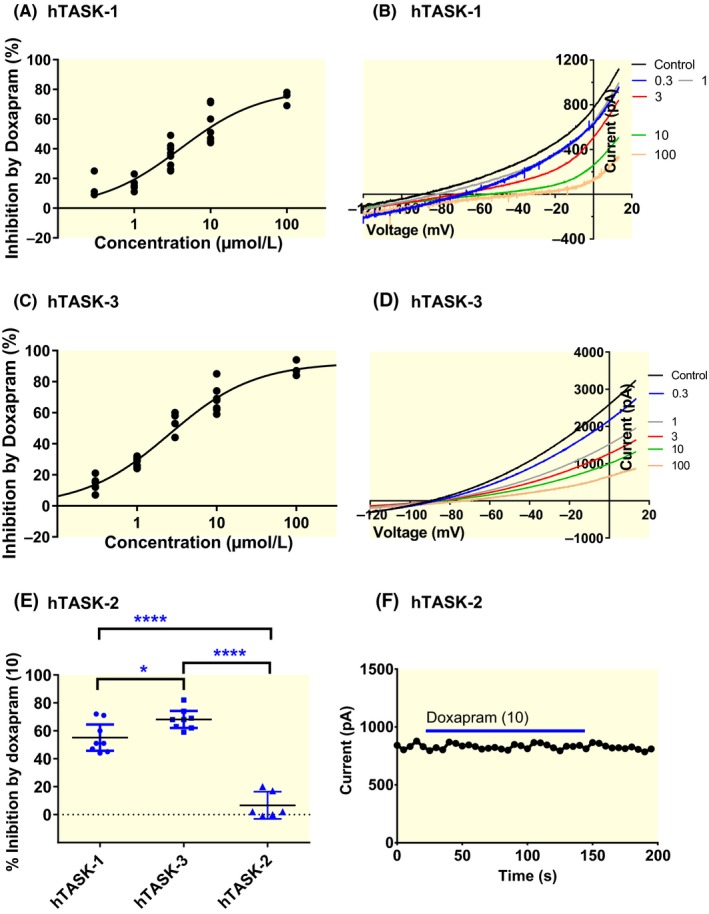
Effect of doxapram on human cloned TASK‐1, TASK‐3 and TASK‐2 channels. (A) Concentration‐response curve for doxapram inhibition of human (h) TASK‐1 current. (B) hTASK‐1 currents evoked by ramp changes in voltage in control conditions and in the presence of doxapram over a range of concentrations (0.3‐100 µM). (C) Concentration‐response curve for doxapram inhibition of hTASK‐3 current. (D) hTASK‐3 currents evoked by ramp changes in voltage in control conditions and in the presence of doxapram over a range of concentrations (0.3‐100 µM). (E) A plot of % inhibition by 10 µM doxapram from individual cells expressing either hTASK‐1, hTASK‐3 or hTASK‐2 human cDNA. Error bars represent the 95% CI. *P < .05, ****P < .0001; One‐way ANOVA, followed by a Dunnett's multiple comparisons test. (F) Time course plot showing the acute application of 10 µM Doxapram (blue line) on hTASK‐2 current. Each point is a 5 millisecond (ms) average of the difference current between that at −40 mV and that at −80 mV (see methods for detailed description of voltage‐ramp protocol)

Our initial experiments sought to determine the pharmacological profile of doxapram on homodimeric TASK‐1 and TASK‐3 channels and a structurally related channel from the TALK subfamily, TASK‐2, using cloned human channels, transiently expressed in tsA201 cells and studied using whole‐cell patch clamp electrophysiology. Surprisingly, unlike for cloned rat TASK channels,^15^, ^22^ doxapram showed an increase potency against human TASK‐3 channels, over a range of concentrations (0.3‐100 µM), with a calculated 50% effective concentration (EC_50_) of 2.5 µM [95% CI: 1.9‐3.5] and a Hill slope of 0.8 [95% CI: 0.5‐1.1] (Figure 1). For human TASK‐1 channels the potent inhibitory effect remained similar to rodent channels, with an observed EC_50_ of 4.0 µM [95% CI: 2.1‐7.9] and a Hill slope of 0.8 [95% CI: 0.7‐1.0] for TASK‐1 (Figure 1). Similar to effects seen with rodent channels,^15^ recovery from inhibition of TASK‐1 by doxapram (10 µM) was slow and mostly incomplete, whilst for TASK‐3, recovery from doxapram (10 µM) inhibition was faster and more complete. By contrast, doxapram (10 µM) had little effect on currents through TASK‐2 channels (Figure 1E,F). Acute application of 10 µM doxapram to WT TASK‐2 resulted in an inhibition of 7% [95% CI: −3 to 16; n = 6], which was significantly less (*P* < .05, one‐way ANOVA, followed by a Dunnett's multiple comparisons test, [95% CI of difference: 35‐61]) than the 55% inhibition [95% CI: 45‐65; n = 8] seen for TASK‐1 channels and also significantly less [*P* < .05, 95% CI of difference: 48‐75] than the 68% [95% CI: 62‐74; n = 8] inhibition seen for TASK‐3 channels.

### 2.2 Doxapram is a more potent inhibitor of mouse TASK‐1 than of mouse TASK‐3 channels and mouse TASK‐3/‐1 heterodimer channels

**Figure 2:**
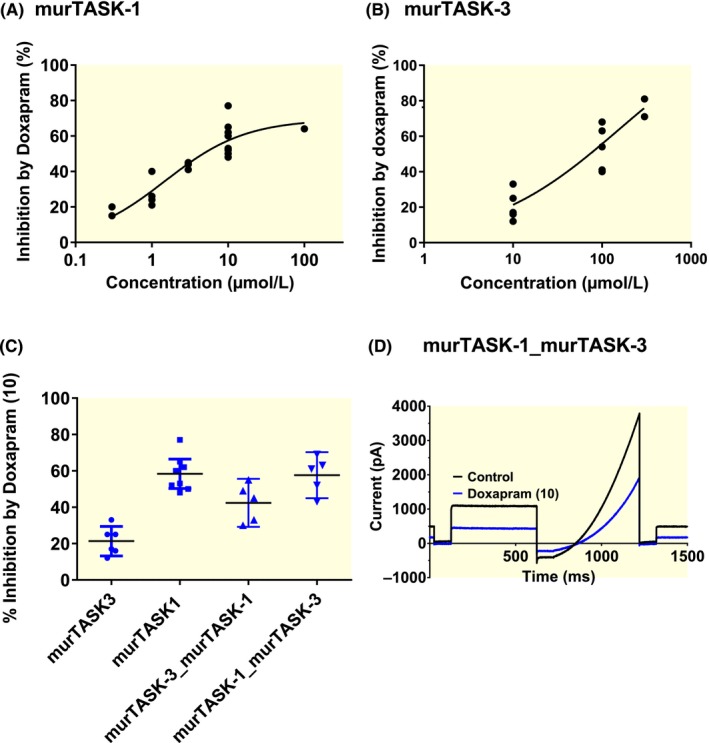
Effect of doxapram on mouse cloned TASK‐1, TASK‐3 homodimeric and forced heterodimeric channels. (A) Concentration‐response curve for doxapram inhibition of murTASK‐1 current over a range of concentrations (0.3‐100 µM). (B) Concentration‐response curve for doxapram inhibition of murTASK‐3 current over a range of concentrations (10‐300 µM). (C) A plot of % inhibition by 10 µM doxapram from individual cells expressing either homodimeric murTASK‐3, homodimeric murT1, heterodimeric murTASK‐3_murTASK‐1 and heterodimeric murTASK‐1_murTASK‐3 cDNA. Error bars represent the 95% CI. (D) Raw data trace from exemplar mouse heterodimeric murTASK‐1_murTASK‐3 in control (black line) and 10 µM doxapram (blue line) using a step‐ramp voltage protocol as detailed in the Methods

As the effect of doxapram was different to that demonstrated on cloned rat channels,^15^ we looked to determine whether the difference observed with human cloned channels was a species‐dependent effect. To do this, we studied the effect of doxapram on cloned mouse channels over a range of concentrations. Similar to cloned rat TASK‐1 and TASK‐3 channels, TASK‐1 and TASK‐3 channels from mouse (mur) were inhibited differentially by doxapram. Acute application of doxapram to murTASK‐1 channels at a concentration of 1, 3 and 10 µM resulted in current inhibition of 28% [95% CI: 14‐41, n = 4], 43% [95% CI: 38‐49, n = 3] and 58% [95% CI: 50‐66, n = 8], respectively, with a calculated 50% effective concentration of 1.5 µM [95% CI: 0.6‐3.9] (Figure 2A). For murTASK‐3, acute application of doxapram at concentrations of 10 and 100 µM resulted in current inhibition of 21% [95% CI: 13‐29, n = 6] and 56% [95% CI: 42‐69, n = 6], respectively, with an estimated 50% effective concentration ≥100 µM, around 100‐fold higher than seen for murTASK‐1 channels (Figure 2B). The inhibitory effect of doxapram was significantly smaller at 10 µM for murTASK‐3 [*P* < .05, 95% CI of difference: 27‐48], compared to murTASK‐1, as was observed with rat TASK channels.^15^

As the predominant channel formed in rat carotid bodies are heterodimers of TASK‐1 and TASK‐3,^13^, ^14^ we also tested the effect of doxapram on forced heterodimers of murTASK‐1 and 3 channels. Combining murTASK‐1 channels with murTASK‐3 channels, conferred sensitivity to doxapram. Doxapram (10 µM) inhibited murTASK‐3/murTASK‐1 heterodimer channels by 42% [95% CI: 29‐56; n = 5], which was an intermediary effect between homodimeric murTASK‐1 (58% [95% CI: 50‐66; n = 8]) and homodimeric murTASK‐3 (21% [95% CI: 13‐29; n = 6] Figure 2C). Interestingly doxapram (10 µM) inhibited the reverse murTASK‐1/murTASK‐3 heterodimer channels by 58% [95% CI: 45‐70; n = 5, Figure 2C,D], which was similar to effects seen on homodimeric murTASK‐1 channels (*P* > .05 [95% CI of difference: −14 to 16]).

### 2.3 The Carboxy terminal domains are involved in transducing the effect of doxapram

**Figure 3:**
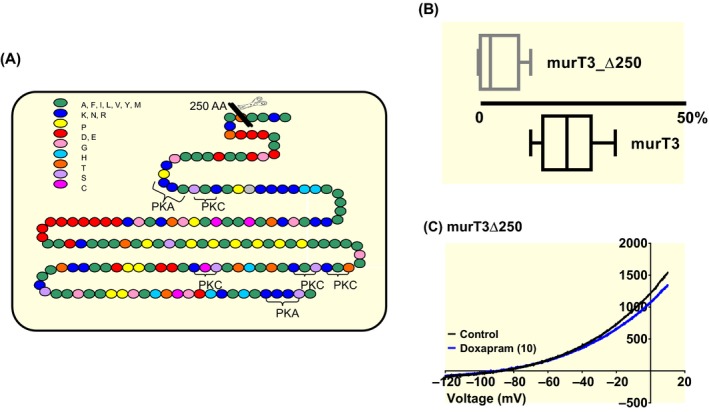
Removal of the carboxy terminal of murTASK‐3 (murT3) attenuates doxapram effect further. (A) Cartoon to depict the amino acid structure of the carboxy terminal of murT3, the location of putative phosphorylation sites and the introduction of the stop codon. (B) Box and Whiskers plot of doxapram (10 µM) inhibition of murT3_Δ250 and murT3 wild type. Bars represent the min and max inhibition for each channel type. (C) murT3_ Δ250 currents evoked by ramp changes in voltage from −120 to + 20 mV in control conditions (black line) and in the presence of 10 µM doxapram (blue line)

The difference in sensitivity to doxapram seen between rodent TASK‐1 and TASK‐3 channels was suggested to occur, although not exclusively, because of structural differences between the carboxy terminals of the channels.^15^ MurTASK‐1 and TASK‐3 channels share only 50% identity, with the least identity occurring in their intracellular carboxy terminal domains. MurTASK‐3 channels have many of their regulatory sites, including phosphorylation sites located in their C‐terminal domains (Figure 3A). Removal of the C‐terminus from murTASK‐3 by the incorporation of a stop codon at position 250 (Δ250), resulted in a functional channel, but with a significantly (*P* < .05, [95% CI of difference: −141 to −91], unpaired t test) reduced current of 18 pA pF^‐1^ [95% CI: −1 to 36, n = 5], compared to an average WT current of 134 pA pF^‐1^ [95% CI: 110‐158, n = 5], when transfecting 125 ng µL^−1^ of DNA. Inhibition of this reduced current by doxapram (10 µM) was also severely attenuated (*P* < .05 [95% CI of difference: −27 to −8]) compared to WT (Figure 3B,C). Doxapram (10 µM) inhibited murT3_ Δ250 by 4% [95% CI: −3 to 10, n = 5, Figure 3B].

### 2.4 The Carboxy terminal domains of human TASK‐1 and TASK‐3 are also structurally different, but the effect of doxapram is equal

**Figure 4:**
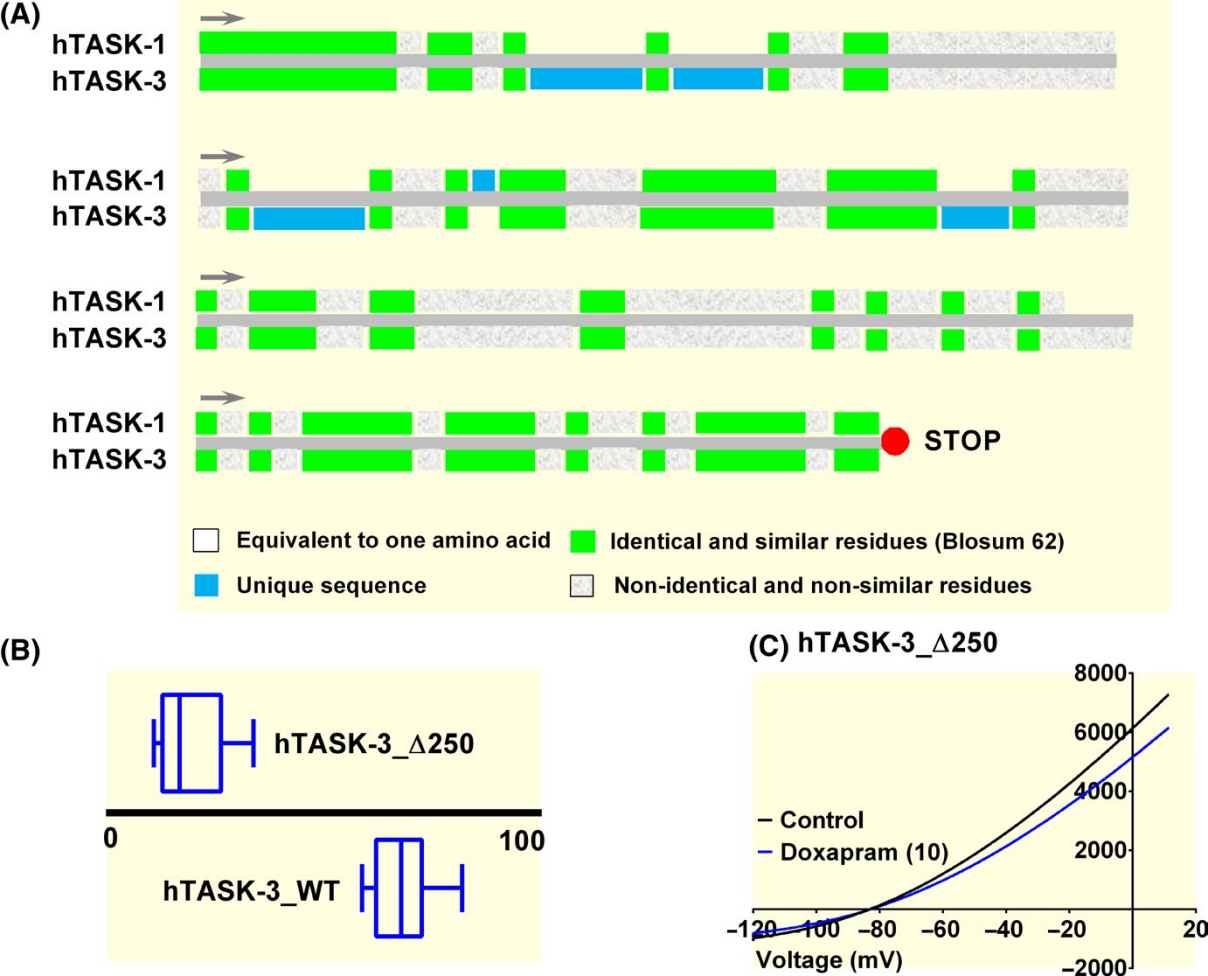
Removal of the carboxy terminal of human TASK‐3 attenuates doxapram effect. (A) Cartoon to compare the identity of the amino acid structure of the carboxy terminal of human TASK‐3 and human TASK‐1. (B) Box and Whiskers plot of doxapram (10 µM) inhibition of hTASK‐3_Δ250 and WT hTASK‐3. Bars represent the min and max inhibition for each channel type. (C) hTASK‐3_ Δ250 currents evoked by ramp changes in voltage from −120 to + 20 mV in control conditions (black line) and in the presence of 10 µM doxapram (blue line)

This difference in effect by doxapram on rodent channels, if explained by their structurally different carboxy terminals, cannot, however, be easily translated to human channels as the effect of doxapram on both channels is similar. Like rodent channels, human TASK‐1 and TASK‐3 channels share 58% identity, with the majority of the structural dissimilarity occurring at the carboxy terminals of the channels (see Figure 4A). As seen for murTASK‐3, truncating the channel to remove the C‐terminal of the human TASK‐3 channel (hTASK‐3_Δ250) significantly attenuated the effect of doxapram (*P* < .05 [95% CI of difference: −57 to −41], n = 8), with inhibition by 10 µM doxapram reduced to 19% [95% CI: 12‐26] (Figure 4B,C). For human TASK‐3, truncating the C‐terminus of TASK‐3, significantly (*P* < .05, [95% CI of difference: 40‐87, unpaired *t* test) increased current to 119 pA pF^−1^ [95% CI: 88‐151, n = 8], compared to an average WT current of 56 pA pF^−1^ [95% CI: 46‐65, n = 16], when transfecting 500 ng µL^−1^ of DNA. Interestingly, current recorded through human TASK‐3 channels in these experimental conditions is almost eightfold smaller than is observed for murTASK‐3 channels.

### 2.5 Mutation of identified amino acids in the pore region of human TASK channels increased channel currents and reduced the efficacy of doxapram

**Figure 5:**
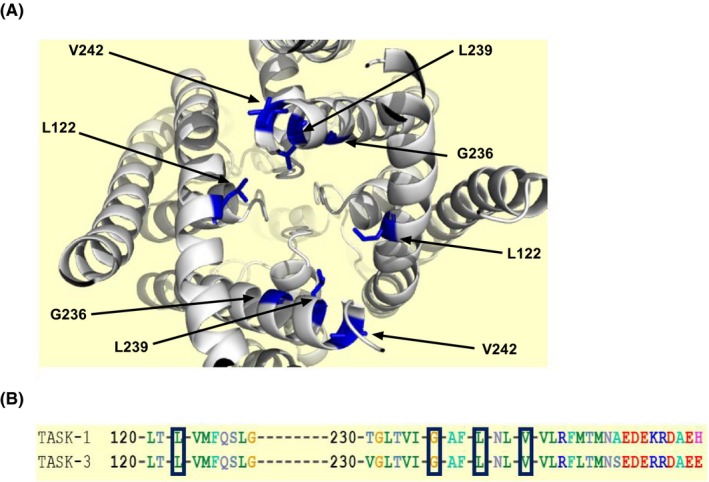
Computer homology model of human TASK‐3 channel with indicated putative binding site. (A) Homology model of human TASK‐3 channel based upon TRAAK crystal structure (PDB ID 3UM7)^54^ depicting location of the four amino acids (AA) that form the putative site, L122, G236, L239 and V242 (shown with arrows) as viewed from beneath the channel. (B) AA sequence alignment of human TASK‐1 and TASK‐3. Dashes represent gaps in the sequence and numbers represent the position of the starting AA. The black box highlights the AA’s that form the putative site

Evidence from molecular modelling, docking and aspartate scanning mutagenesis of rat TASK‐1 and TASK‐3, using inhibitory compounds such as A1899, PKTHPP and doxapram have suggested a common intracellular binding site, comprised of hydrophobic residues from the M2 and M4 transmembrane domains, within the intracellular pore region of these channels.^20^, ^21^, ^22^, ^29^ The key amino acids (AA) identified are Leucine (L) 122, Glycine (G) 236, L239 and Valine (V) 242, which are homologous in the human clones (Figure 5A,B). In rat TASK‐3 mutation of these specific AA to aspartate (D), considerably affected the efficacy of these compounds highlighting the importance of this region for the action of these particular compound types.^22^

**Figure 6:**
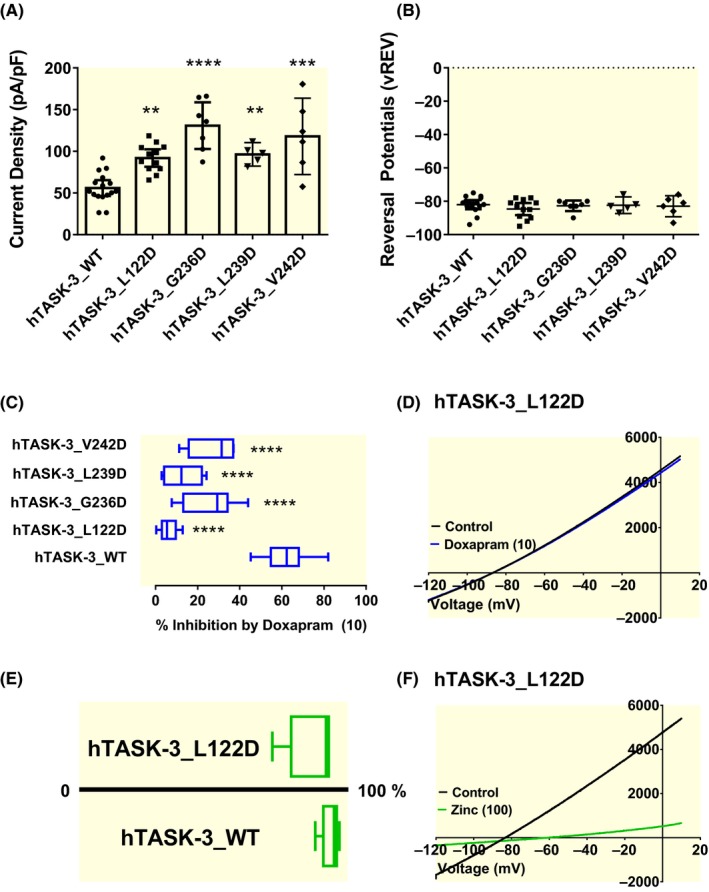
Pore‐lining residues of the M2 and M4 domains are influenential for doxapram inhibition of the human TASK‐3 channel. (A) graph of current density (pA pF‐1) measured from individual cells transiently expressing WT TASK‐3, TASK‐3_L122D, TASK‐3_G236D, TASK‐3_L239D, TASK‐3_V242D. Error bars represent the 95% CI and * statistical significance (*P < .05; ****P < .0001). (B) A plot of zero current level (mV) measured from individual cells transiently expressing WT TASK‐3, TASK‐3_L122D, TASK‐3_G236D, TASK‐3_L239D, TASK‐3_V242D. Error bars represent the 95% CI. (C) Box and Whiskers plot of doxapram (10 µM) inhibition of WT TASK‐3, TASK‐3_L122D, TASK‐3_G236D, TASK‐3_L239D and TASK‐3_V242D. Bars represent the min and max inhibition for each channel type. (D) hT3_ L122D currents evoked by ramp changes in voltage from −120 to + 20 mV in control conditions (black line) and in the presence of 10 µM doxapram (blue line). (E) Box and Whiskers plot of zinc (100 µM) inhibition of WT TASK‐3 and TASK‐3_L122D. Bars represent the min and max inhibition for each channel type. (F) hT3_ L122D currents evoked by ramp changes in voltage from −120 to + 20 mV in control conditions (black line) and in the presence of 100 µM zinc (grey line)

We introduced each of the identified AA into human TASK‐3 and characterized currents mediated through each homodimeric mutated channel (L122D, G236D, L239D and V242D). Mutation of either a hydrophobic leucine (L) or a small uncharged glycine (G) residue to a charged aspartate residue (D), resulted in functional channels with significantly (*P* < .05, one‐way ANOVA followed by a Dunnett's multiple comparisons test) increased currents compared to WT (Figure 6A). The average whole‐cell current measured as a difference between current seen at −40 mV and −80 mV was: 56 pA pF^−1^ [95% CI: 46‐66, n = 15] for WT; 92 pA pF^−1^ [95% CI: 81‐102, n = 12] for L122D; 131 pA pF^−1^ [95% CI: 103‐159, n = 7] for G236D; 96 pA pF^−1^ [95% CI: 82‐111, n = 5] for L239D; and 118 pA pF^−1^ [95% CI: 72‐164, n = 6] for V242D, compared with an average whole‐cell current of 2 pA pF^−1^ [95% CI: 1.5‐2.3, n = 5] for cells only transfected with green fluorescent protein (GFP), data not shown. All mutated currents were outwardly rectifying, with a mean zero current potential of −85 mV [95% CI: −88 to −81, n = 12] for L122D; −83 mV [95% CI: −86 to −80, n = 7] for G236D; −82 mV [95% CI: −87 to −77, n = 5] for L239D) and −83 mV [95% CI: −89 to −77, n = 6] for V242D, compared with −31 mV [95% CI: −43 to −20, n = 5] for GFP‐only cells. None of the zero current potentials for the mutant channels were significantly different (*P* > .05) from WT which was −82 mV [95% CI: −85 to −79, n = 15] (Figure 6B). We then tested whether these mutations modified the potency of doxapram, as had been observed in rat channels.^22^ All four mutations significantly reduced (*P* < .05) doxapram potency, with the largest reduction in effect observed with the L122D mutation (Figure 6C,D). The percentage inhibition was 6% [95% CI: 3‐9, n = 10] for L122D; 26% [95% CI: 15‐36, n = 8] for G236D; 13% [95% CI: 6‐20, n = 8] for L239D; and 27% [95% CI: 13‐41, n = 5] for V242D compared to 62% [95% CI: 57‐67, n = 16] for WT. To further confirm the consensus that doxapram acts within the intracellular pore of TASK channels,^15^ we studied the effect of another well‐known TASK‐3 inhibitor, zinc, which is proposed to have its mode of action from the extracellular side of the channel.^30^, ^31^ Using the aspartate mutant channel, L122D, we found that the effect of zinc, was not affected (*P* > .05 [95% CI of difference: −17 to 3]) by this pore mutation, with 100 µM zinc giving an inhibition of 87% [95% CI: 76‐98, n = 5], compared with 94% [95% CI: 90‐98, n = 5] for WT TASK‐3 (Figure 6 E,F).

### 2.6 Enantomeric separation of doxapram reveals a stereoselective effect on TASK channels

**Figure 7:**
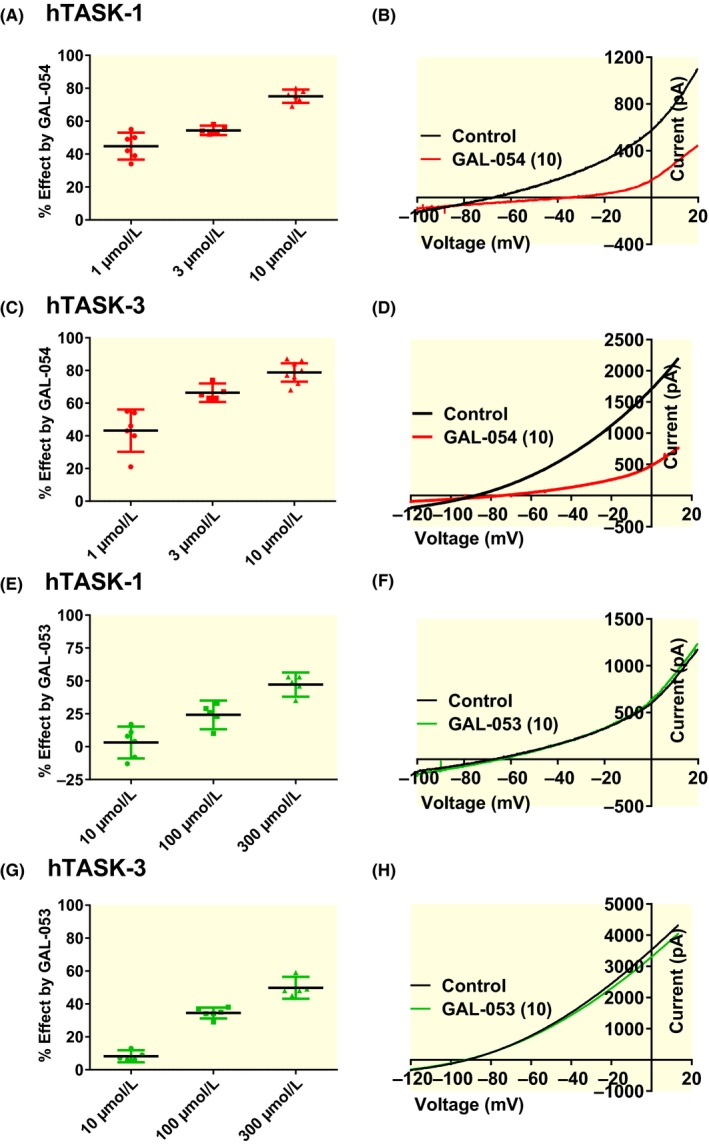
The (+)—enantiomer (GAL‐054) is responsible for the inhibitory effects observed with doxapram on human TASK channels. (A) A plot of % inhibition of 1, 3 and 10 µM GAL‐054 on human TASK‐1 channels. Each point represents an individual cell and the error bars represent the 95% CI. (B) human TASK‐1 currents evoked by ramp changes in voltage from −120 to + 20 mV in control conditions (black line) and in the presence of 10 µM GAL‐054 (red line). (C) A plot of % inhibition of 1, 3 and 10 µM GAL‐054 on human TASK‐3 channels. (D) human TASK‐3 currents in control conditions (black line) and in the presence of 10 µM GAL‐054 (red line). (E) A plot of % inhibition of 10, 100 and 300 µM GAL‐053 on human TASK‐1 channels. (F) human TASK‐1 currents in control conditions (black line) and in the presence of 10 µM GAL‐053 (green line). (G) A plot of % inhibition of 10, 100 and 300 µM GAL‐053 on human TASK‐3 channels. (H) human TASK‐3 currents in control conditions (black line) and in the presence of 10 µM GAL‐053 (green line). The specificity observed with mouse TASK channels remained unchanged with for the (+)—enantiomer GAL‐054, with the enantiomer being twice as potent on murTASK‐1 channels compared with murTASK‐3 (data not shown). A concentration of 10 µM inhibited murTASK‐1 channels by 63% [95% CI = 61 to 65, n = 3] compared to an inhibition of 33% [95% CI = 19 to 46, n = 4] for murTASK‐3

Doxapram is a racemic compound that can be chirally separated into a (+)—enantiomer (GAL‐054) and (−)—enantiomer (GAL‐053) (Galleon Pharmaceuticals Inc).^27^ The eutomer (GAL‐054) was found to be a superior respiratory stimulant compared to doxapram when tested in animal models, whilst the distomer (GAL‐053) was markedly inferior.^24^, ^25^, ^26^ We looked to see what effect these separate enantiomers of doxapram had on human TASK‐1 and TASK‐3 current. Compared with doxapram and GAL‐053, GAL‐054 was twice as potent as doxapram with EC_50_s of ~1.6 and 1.4 µM for human TASK‐1 and TASK‐3 respectively. GAL‐053 had reduced potency on TASK‐1 and TASK‐3, with EC_50_s ~336 and 286 µM respectively. Acute application of 1, 3 and 10 µM GAL‐054 to human TASK‐1 expressing tsA201 cells, resulted in an inhibition of 45% [95% CI: 37‐53, n = 6]; 54% [95% CI: 52‐57, n = 5]; and 75% [95% CI: 71‐79, n = 6] respectively. For human TASK‐3 inhibitions of 43% [95% CI: 30‐56, n = 6]; 66% [95% CI: 61‐72, n = 5]; and 79% [95% CI: 73‐84, n = 8] were seen for the same concentrations (Figure 7A‐D). Acute application of 10, 100 and 300 µM GAL‐053 to human TASK‐1, resulted in an inhibition of 3% [95% CI: −9 to 15, n = 6]; 24% [95% CI: 13‐35, n = 5] and 47% [95% CI: 38‐56, n = 5]. For human TASK‐3 inhibitions of 8% [95% CI: 5‐12, n = 6]; 35% [95% CI: 31‐38, n = 6] and 50% [95% CI: 43‐56, n = 5] were seen for the same concentrations (Figure 7E‐H).

## 3 DISCUSSION

Doxapram is one of the few respiratory stimulants still in clinical use and has been shown to inhibit cloned rat TASK channels and native TASK channels (TASK‐1/3 heterodimers) in rat type 1 cells^5^, ^15^, ^16^, ^22^ with highest potency observed for rat TASK‐1 (TASK‐1 > TASK‐1/3 heterodimer > TASK‐3).

The mode of action by which doxapram acts as a respiratory stimulant in humans is controversial. In part, this is a consequence of differing effects observed in various animal models. In particular, the stated molecular mechanism by which doxapram has its effect, has been characterized entirely in rodents.^5^ In this study, we characterized the effect of doxapram on cloned human TASK channels (TASK‐1 and TASK‐3), to give a better understanding of the pharmacological profile of this drug in humans, and any potential clinical consequences for patients.

Interestingly, we have found that doxapram is an equally potent inhibitor of both human TASK‐1 and TASK‐3 channels, which is different to that seen previously in rat channels, where the drug is 90‐fold more potent on TASK‐1 or heteromultimers of TASK‐1 and TASK‐3 than on TASK‐3.^15^, ^22^ We investigated whether this was a species dependent effect by repeating the same experiments on cloned mouse TASK channels and found, as for rat channels, that doxapram was a more potent inhibitor of mouse TASK‐1 channels and heteromultimers of these channels, than mouse TASK‐3 channels.

The increased potency of doxapram for TASK‐3 channels may not alter the determined molecular mechanism by which doxapram has its effect on TASK channels in the carotid bodies, as heteromultimers of TASK‐1 and TASK‐3 are the predominant channel in these type 1 cells, at least for rodents.^13^, ^14^ Its inhibitory effect on human TASK‐3 channels may however, contribute to the side effect profile of this compound, in humans. For example, changes in systolic blood pressure have been linked to changes in TASK‐3 channel expression.^32^ Indeed, some of the major limitations to doxapram's clinical use has been its analeptic and pressor effects, which includes an increase in arousal, panicogenic activity, increased hyperventilation, increased blood pressure and heart rate, and convulsions, in patients.^27^, ^33^ Many of these side effects are observed primarily in patients that already suffer from these conditions and typically are manifest during continuous intravenous infusion, due the compounds short half‐life.^34^

We also show that the effect of doxapram is restricted to the TASK family of channels, with the compound having no effect on a closely related channel member, TASK‐2. However, inhibitory effects have been observed with TASK‐2 at much higher concentrations of doxapram (300 µM and 1 mM).^15^

Removing the C‐terminus of TASK‐3 channels, both for murine and human TASK‐3 channels, significantly reduces the effectiveness of doxapram (Figures 3B and 4B). This correlates with earlier experiments in rat cloned channels, where C‐terminal domain swopping of TASK‐1 to TASK‐3, increased or decreased the sensitivity of the channels to doxapram^15^ and reduced the effectiveness of the endocannabinoid, methanandamide.^35^ The rationale for the earlier experiments, was because of the high sequence dissimilarity between rodent TASK‐1 and TASK‐3 C‐termini. However, for human channels, the potency of doxapram is equal, despite the same high sequence dissimilarity in the C‐termini between the two channels (Figure 4A), making the importance of the C‐termini harder to interpret. Because of the importance of this region for signalling inputs, such as phosphorylation,^36^ one can hypothesize that the phosphorylation state of the channel may be important for doxapram's effect, particularly as the number of putative phosphorylation sites are different between TASK‐1 and TASK‐3, with more predicted sites on mouse and human TASK‐3.^37^ For human TASK‐3 channels, C‐terminal truncation resulted in a two‐fold increase in current. By truncating the channel, it is possible that the channel has been pushed into an open state, perhaps by locking the channel into a particular phosphorylation state, or because gating at the selectivity filter is disrupted by C‐terminus truncation. Indeed, for known gain‐of‐function mutations on these channels and other related K2P channels, regulation by blockers or activators is modified when the link between the C‐terminus and the selectivity filter is disrupted.^37^, ^38^ Surprisingly, however, truncation of murTASK‐3 channels, resulted in a significant reduction in current recorded through these channels, but this still attenuated the effect of doxapram. With the structure of the intracellular C‐termini of K2P channels not determined by existing crystal structures, the involvement of this region remains difficult to evaluate. The C‐terminal domain of another potassium channel, GIRK2, has been resolved by crystallography and this shows that for this particular channel, the C‐terminal acts as an additional gate to regulate pore access.^39^

Recent molecular modelling studies of rat TASK‐1 and rat TASK‐3 channels using TASK selective inhibitory compounds such as A1899, PKTHPP, ML365 and doxapram, have suggested a common intracellular binding site, within the pore region of these channels.^20^, ^21^, ^22^ To further elucidate the molecular mode of action of doxapram on human channels, we examined previously identified hydrophobic residues in rat, located on the M2 and M4 transmembrane regions of TASK‐3, that face the pore region of the channel. These residues are suggested to form a common intracellular binding site for a number of respiratory stimulants, including doxapram. In particular, mutation of a leucine (L) on the M2 region, L122, and another on the M4 region, L239, significantly attenuates doxapram effect. It has previously been suggested that L122 and L239, create a hydrophobic narrowing of the pore, which affects the potency of three breathing stimulants, PKTHPP, A1899, and doxapram.^22^ When these normally hydrophobic residues are mutated to hydrophilic (lipid‐repelling) residues, such as aspartate residues, this creates a barrier in the pore, because of either an increase in water and potassium ion occupancy in the pore and/or this region acts as a fulcrum point for channel gating, preventing access of the drug.^22^ This correlates with another previous molecular modelling study of doxapram, which suggests the drug has a high affinity for a hydrophobic cleft in which to bind^40^ and with a study conducted on TASK‐3_L122D, which suggested that mutating this amino acid to an aspartate produces a fixed open conformation that reduces the effects of anaesthetics.^29^ As well as for doxapram^22^ the L239 residue has previously been identified in TASK‐1 for its involvement in A1899 inhibition^20^ and more recently in A293 inhibition.^41^

In our experimental conditions, the currents recorded through all four mutant channels (L122D, G236D, L239D and V242D) are significantly larger than for WT channels, suggesting that the channel may be gated into an open confirmation, reducing the efficacy of inhibitors, such as doxapram, similar to that seen with the T3_Δ250 channel.

For L122D in particular, the mutation that caused the most dramatic reduction in doxapram effect, it has recently been shown that D or N mutations at this position in all K2P channels act as gain of function mutations^42^ and mutations at this position alter the effectiveness of a number of K2P channel regulators.^42^, ^43^ This may confound interpretation of functional experiments that suggest this residue is involved in regulator binding. To address this, we investigated whether this particular mutation attenuated the effect of another known TASK‐3 antagonist, zinc, which has been shown to act on residues on the extracellular side of the channel.^30^, ^31^ This mutation had no effect on zinc inhibition of the channel, which is in agreement with the hypothesis that doxapram does indeed act at a site within the intracellular pore region of the channel.^22^

Since the submission of this manuscript a crystal structure of TASK‐1, bound to inhibitory compounds, was released.^44^ The resolved structure, revealed the presence of a unique gate, termed “X‐gate”, at the intracellular entrance to the vestibule, formed from a conformational rearrangement of the M4 helices, involving the VLRFMT region. For two novel inhibitory compounds (BAY 1000493 and BAY 2341237) it was shown that the compounds bind within an inner vestibule, directly below the selectivity filter. Consistent with previously published work,^20^, ^22^, ^40^, ^41^ L122 and L239, along with some other amino acids, were important for binding and trapping of these inhibitory compounds, within TASK‐1 channels. In particular, they found that L122 projected into the vestibule below the compounds, holding them in place and this was thought to be responsible for the slow compound washout rates observed for these and others compounds.^15^ Indeed, we also observed that for TASK‐1, washout of doxapram, was slow and often incomplete, however, this was not true for TASK‐3. This suggests that despite high sequence similarity between TASK‐1 and TASK‐3 in this region, the arrangement of amino acids within the vestibule and the X‐gate (VLRFMT for TASK‐1, but VLRFLT for TASK‐3) may differ slightly between the two channels and perhaps also for heterodimers of the channels.

Interestingly, another putative doxapram binding residue, G236, is mutated to a large positively charged arginine (R) in a condition known as Birk Barel Mental Retardation Syndrome or more recently, KCNK9 Imprinting syndrome.^23^, ^45^ In this homodimeric conformation the channel is poorly functioning, with low current levels, which are inwardly rectifying and show altered potassium selectivity.^46^ When G236 is mutated to an aspartate, however, the current is significantly increased through these channels, remains outwardly rectifying and potassium selective.

We have also shown that chiral separation of doxapram into its positive and negative enantiomers, results in a highly potent inhibitory eutomer (GAL‐054), which was twofold more potent than doxapram and a poorly inhibitory distomer (GAL‐053) when tested on human cloned channels, transiently expressed in a human tsA201 cells. These data correlate nicely with pre‐clinical data in opioid challenged rats and cynomolgus monkeys, where GAL‐054, dose‐dependently increased minute volume when administered intravenously, whilst GAL‐053, had no effect.^24^, ^25^, ^26^ It was also observed in these studies that some of the known side‐effects of this drug, were restricted to the distomer, raising hope for an improved ventilatory stimulant, with few side effects. Disappointingly, the known pressor effects of doxapram in human and dogs^47^, ^48^ were still evident with GAL‐054. In conscious rats and in healthy human volunteers of a Phase 1 clinical trial. GAL‐054 was found to increase blood pressure by 15%‐20% (unpublished data, Galleon Pharmaceuticals)^27^ and consequently was no longer pursued because of patient safety concerns. It has been suggested that this observed increase in blood pressure may occur because of an increase in catecholamine levels during administration of doxapram.^49^ As the proposed mechanism of action by which doxapram reverses respiratory depression is via the direct stimulation of peripheral chemoreceptors of type 1 cells within the carotid bodies, resulting in a subsequent release of catecholamines.^4^, ^50^ It remains possible that any drugs that target TASK channels in carotid bodies, may also increase blood pressure. It should also be borne in mind that the hemodynamic reflex response to selective stimulation of carotid body chemoreceptors is complex and often described as context specific, and that evoked ventilatory stimulation can be associated with early pressor and later depressor effects.^51^, ^52^, ^53^

In our study, all experiments were performed using human cell lines in normoxic conditions at room temperature. Whilst this gives consistency of basal responses on which to measure the effects of doxapram, it is also a limitation as we did not investigate the effects of doxapram under conditions of hypoxia, which causes an inhibition of both TASK‐1 and TASK‐3 channels, or at normal body temperature. In clinical conditions, doxapram is often used in hypoxic conditions and always at body temperature, which makes it less easy for us to extrapolate effects seen in our controlled cell systems to clinical situations.

Nevertheless, the data reported here showing that the effect on TASK channels is restricted to the positive enantiomer; the differential potencies between human and murine channels and the molecular information regarding a potential intracellular binding site within the pore region of TASK channels, will be of benefit for the design of new therapeutic molecules with higher potency, higher specificity and fewer associated side effects.

## 4 MATERIALS AND METHODS

### 4.1 Molecular biology

Murine (mur) wild‐type (WT) TASK‐1 (KCNK3, Genbank^TM^ DQ185133) and WT murTASK‐3 (KCNK9, Genbank^TM^ AH009585.2) cDNA were cloned into pCS2^+^ vector and were a kind gift from William Wisden (Imperial College, UK). Human WT TASK‐1 (Genbank^TM^ AF006823.1), WT TASK‐3 (Genbank^TM^ AF212829) and WT TASK‐2 (KCNK5, Genbank^TM^ AF084830.1) cDNA’s, were cloned into pcDNA3.1^+^ vector (Invitrogen) and were a kind gift from Helen Meadows (GlaxoSmithKline).

### 4.2 Mutations

Point mutations (L122D, G236D, L239D, V242D or a stop codon) were introduced by site‐directed mutagenesis into TASK‐3 cDNA using the Quikchange kit (Stratagene) as previously described.^54^

### 4.3 Cell culture

All experiments were performed using a modified human embryonic kidney 293 cell line, tsA201 (ECACC; Sigma‐Aldrich), prepared and maintained as previously described.^54^

### 4.4 Transfection

For the electrophysiological experiments, cells were transiently transfected using a modified calcium phosphate protocol, following a process as previously described.^54^ Vectors cloned with the gene of interest and a similar vector encoding the cDNA for GFP were added to each well at a concentration of 0.5 µg per well, with the exception being for murTASK‐3, where 0.125 µg per well was used.

### 4.5 Whole‐cell patch clamp electrophysiology

Currents were recorded from tsA201 cells transiently transfected with the channel of interest using whole‐cell patch clamp in a voltage clamp configuration and a step‐ramp voltage protocol as previously described.^54^ Briefly, all experiments were conducted at room temperature (20‐24°C) using an external solution composed of 145 mM of NaCl, 2.5 mM of KCL, 3 mM of MgCl_2_, 1 mM of CaCl_2_ and 10 mM of HEPES (pH 7.4, using NaOH) and an intracellular pipette solution composed of 150 mM of KCL, 3 mM of MgCl_2_, 5 mM of EGTA and 10 mM of HEPES (pH adjusted to 7.4 with KOH). External solution and modulatory compounds were superfused at a rate of 4‐5 mL min^−1^. Currents were recorded using an Axopatch 1D patch clamp amplifier (Molecular Devices), filtered at 0.3 kHz, digitized at 1 kHz.

### 4.6 Data analysis and statistics

For analysis of outward current, we measured the current difference between the −80 and −40 mV. The current‐voltage graphs were obtained from the ramp change in voltage between −120 and +20 mV. For each cell, the current amplitude (pA) was normalized to the cell capacitance (pF). The currents obtained were analysed using pCLAMP 10.2 software (Molecular Devices), Microsoft Excel and GraphPad Prism 6 or 7 software. Data were expressed as the mean ± 95% Confidence Intervals (CI), and *n* represents the number of individual cells. For EC_50_ calculations, concentration‐response curves were fitted using the Hill equation. Statistical analysis used were one‐way ANOVA with a post‐hoc Dunnett's test or an unpaired/paired Student's *t* test. Data were considered statistically different if *P* < .05. The data and statistical analysis comply with the recommendations on experimental design and analysis in pharmacology.^55^

### 4.7 Chemicals

Doxapram, GAL‐053 and GAL‐054 were a kind gift of Galleon Pharmaceuticals Inc and were prepared in water to create 10 mM stock solutions that were then diluted, in external solution, to desired concentration just before use. Zinc Chloride was purchased from Sigma‐Aldrich and was made up in water to create a 100 mM stock.

### 4.8 Homology modelling

hTASK‐3 (UniProtKB/Swiss‐Prot ID Q9NPC2) and murTASK‐3 (UniProtKB/Swiss‐Prot ID Q3LS21) homology models were developed as previously described^45^ using Modeller 9v8.^56^ The human TWIK‐related arachidonic acid activated K (TRAAK) structure (PDB ID 3UM7)^57^ was used as a template for TASK‐3 modelling, ClustalW^58^ was used to align the TRAAK and TASK‐3 sequences.

## CONFLICTS OF INTEREST

The authors declare that they have no conflicts of interest. We confirm that the material submitted conforms to Good Publishing Practice in Physiology: Good publication practice in physiology.^59^
